# Plasma Levels of IL-37 and Correlation with TNF-α, IL-17A, and Disease Activity during DMARD Treatment of Rheumatoid Arthritis

**DOI:** 10.1371/journal.pone.0095346

**Published:** 2014-05-01

**Authors:** Ping-Wei Zhao, Wei-Guang Jiang, Li Wang, Zhen-Yu Jiang, Yu-Xing Shan, Yan-Fang Jiang

**Affiliations:** 1 Key Laboratory of Zoonosis Research, Ministry of Education, The First Hospital, Jilin University, Changchun, China; 2 Department of Rheumatism, The First Hospital, Jilin University, Changchun, China; Virginia Tech, United States of America

## Abstract

The aim of this study was to assess the change of IL-37 concentrations in rheumatoid arthritis (RA) patients under Disease-modifying anti-rheumatic drug (DMARD) therapy, and to establish a correlation between Interleukin-37 and pro-inflammatory cytokines in plasma and disease activity. The plasma level of IL-37 was determined using ELISA in 50 newly diagnosed RA patients and 30 healthy controls (HC). Plasma levels of IL-17A, IL-6 and TNF-α were measured using flow a cytometric bead array assay. We found that the concentrations of IL-37, as well as IL-17A, IL-6 and TNF-α, were higher in plasma of RA patients compared to HCs. Compared to patients who did not respond to DMARD treatment, treatment of patients responsive to DMARDs resulted in down-regulation of IL-17A, IL-6 and TNF-α expression. The plasma level of the anti-inflammatory cytokine IL-37 was also decreased in drug responders after DMARD treatment. The plasma level of IL-37 in RA patients was positively correlated with pro-inflammatory cytokines (IL-17A, TNF-α) and disease activity (CRP, DAS28) in RA patients. IL-37 expression in RA and during DMARD treatment appears to be controlled by the level of pro-inflammatory cytokines. This results in a strong correlation between plasma levels of IL-37 and disease activity in RA patients.

## Introduction

Rheumatoid arthritis (RA) is a chronic inflammatory disorder with autoimmune etiology characterized by joint inflammation, T cell infiltration of the synovium, synovial hyperplasia, neoangiogenesis, involvement of many catabolic cytokines, and progressive destruction of articular cartilage and bone [1]. Multiple pro-inflammatory cytokines involved in RA contribute to the proliferation of synovial tissue and joint erosion [2]. We hypothesize that an imbalance between pro-inflammatory cytokines and anti-inflammatory cytokines may be a primary factor in disease progression.

TNF-α is a pivotal pro-inflammatory cytokine in the pathogenesis of RA that is known to induce adhesion molecule and proteinase gene expression, and play a major role in the progression of joint destruction and proliferation of synoviocytes [3]. IL-6 is another pro-inflammatory cytokine with multiple functions, and is believed to play a pivotal role in the inflammatory process, in osteoclast-mediated bone resorption, and in synovitis. In autoimmunity, IL-6 may induce pro-inflammatory T-helper 17 (Th17) cell differentiation and B-cell antibody production [4]–[6]. IL-17A is one of the most important mediators involved in T cell-mediated synovial inflammation and contributes to bone destruction through increase of migration, chemokine gene expression, and invasiveness of synoviocytes in RA [7], [8]. Therefore, therapies targeting these cytokines or their receptors are recognized as effective treatments for patients with RA [9].

IL-37 is a newly defined member of the IL-1 cytokine family, and it is a key cytokine in regulating inflammation [10]. Expression of IL-37 in macrophages or epithelial cells almost completely inhibits the synthesis of pro-inflammatory cytokines [11], [12]. In addition, IL-37 protein can be up-regulated by pro-inflammatory cytokines and inflammatory stimuli [12], [13]. IL-37 acts as an essential inhibitor of inflammation and innate immunity in various diseases [14]. Marcel F Nold. *et al.* have reported that immunohistochemical staining of synovial lining from RA patients revealed greater amounts of IL-37 compared to that of HCs [13]. In this study, we assessed the plasma levels of IL-37 in RA patients, and analyzed the relationship between IL-37 and pro-inflammatory cytokines or disease activity.

Disease-modifying anti-rheumatic drugs (DMARDs) help to ease symptoms and to slow progression of RA. Methotrexate is often the first drug given for rheumatoid arthritis [15]. In this study we combined methotrexate with Leflunomide, another DMARD, and determined the expressions of both pro- and anti-inflammatory cytokines in RA patients during DMARD therapy.

## Materials and Methods

### Patients

Fifty newly diagnosed (<12 months of disease duration) RA patients were recruited sequentially at the inpatient service of the First Hospital of Jilin University from March 2011 to October 2012 and were orally treated weekly with 10 mg methotrexate (MTX, Shanghai Xinyi Pharmacy, Shanghai, China), and daily with 20 mg Leflunomide (Fujian Huitian Pharmacy, Fujian, China) for 90 consecutive days. Thirty age- and sex-matched healthy volunteers were included in this study. Individual patients with RA were diagnosed, according to the diagnosis criteria established by the American College of Rheumatology [16], and the disease severity of individual patients was evaluated using the disease activity score 28 (DAS28) [17]. According to disease severity classification of RA patients, serious disease activity is defined as DAS28>5.1; medium disease activity is defined as 3.2<DAS28<5.1; a fundamentally alleviated pathologic condition is defined as 2.6<DAS28<3.2; and complete improvement is defined as DAS28<2.6. According to the European League Against Rheumatism (EULAR) response criteria, a decrease in the DAS28 score of 0.6 or less is considered a poor response, while a decrease of greater than 1.2 indicates a moderate or good response, dependent on whether an individual’s DAS28 score at the end point is above or below 3.2, respectively. All RA patients included in this study had initial DAS 28 scores of >3.2 during the activity phase of the disease. The first blood sample was collected from patients before the initiation of treatment with DMARDs. Following DMARD therapy, we defined drug-responders as patients whose DAS28 score decreased by more than 1.2 points, while non-responders were defined by a decrease of the DAS28 score by 0.6 points or less. None of the patients had been treated with DMARDs, immunosuppressive, or other drugs, or had other chronic inflammatory or autoimmune diseases prior to the first sampling. None of the patients were treated with NSAIDs or glucocorticoids except for MTX plus leflunomide during RA therapy. Sera of RA patients and healthy controls were stored at –80°C prior to evaluation of cytokines. All subjects signed an informed consent form prior to the initiation of the study, which was approved by the Ethics Committee of the First Hospital of Jilin University.

### Laboratory Tests

Full blood counts and erythrocyte sedimentation rates (ESR) of individual subjects were determined. Levels of serum C-reactive protein (CRP), rheumatoid factor (RF), and anti-cyclic citrullinated peptide (anti-CCP) were determined by scatter turbidimetry using a Siemens special protein analyzer (Siemens Healthcare Diagnostics Products, GmbH, Germany).

### Measurement of IL-37 by ELISA

Plasma level of IL-37 in HCs and RA patients were assayed using a commercially available enzyme-linked immunosorbent assay (ELISA) kit (human IL-37 ELISA, AdipoGen, Switzerland). A polyclonal antibody specific for IL-37 was used for coating of 96-well microtiter plates. Briefly, individual sera were diluted in Diluent 1X at 1∶2. 100 µl of the different standards were added into the appropriate wells in duplicate. 100 µl of diluted plasma were added in duplicate into additional wells. The plate was covered and incubated overnight at 4°C. After extensive washing to remove unbound compounds, IL-37 was recognized by the addition of 100 µl of polyclonal antibody specific for IL-37 (detection antibody). After removal of excess polyclonal antibody, 100 µl HRP-conjugated anti-rabbit IgG (secondary antibody conjugate) was added. Following a final wash, the bound peroxidase activity was quantified using 100 µl of the substrate 3,3′,5,5′-tetramethylbenzidine (TMB). The color reaction was developed at ambient temperature in the dark for 10 minutes. The intensity of the color reaction was measured at 450 nm after acidification and was directly proportional to the concentration of IL-37 in the standards. According to a standard curve established using recombinant IL-37, the concentration of serum IL-37 in individual samples were calculated. The detection range of the IL-37 ELISA kit was 0.016–1 ng/mL.

### Cytometric Bead Arrays of Serum Cytokines

The plasma levels of TNF-α, IL-6, and IL-17A in HCs and RA patients were determined by cytometric bead array (CBA) [18], according to the manufacturer’s protocol (BD Biosciences, San Joes, USA). Briefly, 25 µl of individual sera were used in duplicate for analysis, as described previously [19]. The serum concentrations of cytokines were quantified using the CellQuestPro and CBA software (Becton Dickinson) on a FACSCalibur cytometer (BD Biosciences).

### Statistical Analysis

All data are expressed as median and range unless specified. The difference between the groups was analyzed by Mann-Whitney test and paired Wilcoxon signed rank tests using SPSS 18.0 software for unpaired and paired comparisons, respectively. The relationship between variables was evaluated using the Spearman rank correlation test. A two-side P value <0.05 was considered statistically significant.

## Results

### Demographic and Clinical Characteristics of Study Subjects

Fifty plasma samples from RA patients (41 female and 9 male) (range 32–63 years of age) and 30 samples from age- and sex-matched healthy volunteers were analyzed in this study. ESR, CRP, RF, anti-CCP and DAS28 were higher in patients with RA than in HCs (in all cases P<0.05). Following DMARD therapy for three months, these clinical parameters decreased in patients responding to treatment, compared to the same parameters at base line; however, there was no statistically significant difference between the same parameters in non-responders before and after DMARD treatment (Table 1).

**Table 1:** Demographic and clinical characteristics of subjects.

Group	HCs	Drug responder (n = 34)		Drug-nonresponder (n = 16)	
		Before	After	Before	After
ESR (mm/hour)	13(4–19)	53(28–120)*	21(7–65)* #	69(15–105)*	60(30–95)*
CRP (mg/dl)	4.2(0.3–6.2)	31.6(0.61–141)*	7.6(1.66–40.9)* #	18.9(3.8–104)*	16.4(0.8–93.7)*
RF (IU/ml)	14(0.4–27)	319.4(0.11–1270)*	58 (3.37–501)* #	265.7(25.8–1440)*	163(2.57–1020)*
RF (+/−)	NA	31/3	9/25	15/1	14/2
Anti-CCP (IU/ml)	4.32(0.24–6.29)	547(0.67–2476)*	51.3(5.4–798)* #	652(35.6–2778)*	359(21.9–2250)*
Anti-CCP (+/−)	NA	33/1	7/24	16/0	15/1
DAS28	NA	6.39(3.76–9.85)*	3.38(1.65–5.07)* #	7.15(3.9–10.12)*	5.27(3.74–9.96)*

Data shown are median (range) of each group of subjects. RF: rheumatoid factor; ESR: erythrocyte sedimentation rate; CRP: C-reactive Protein; CCP: cyclic citrullinated peptide; DAS28: Disease Activity Score in 28 joints; NA: not applicable;

*P<0.05 vs. HCs;

# P<0.05 vs. baseline values.

### Plasma Levels of IL-37 in RA Patients during DMARD Treatment

**Figure 1:**
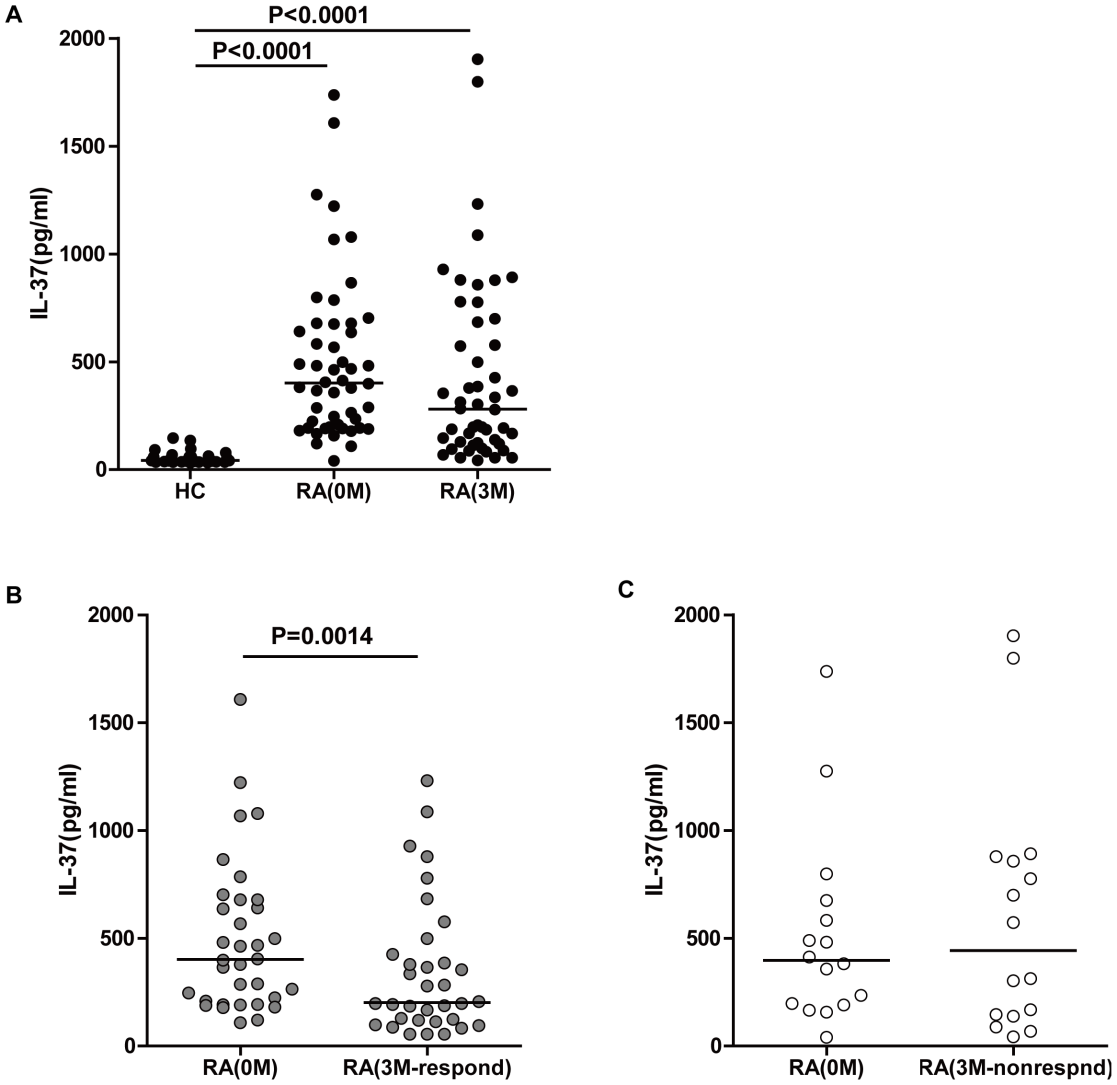
Plasma levels of IL-37 in RA patients. (**A**) Plasma levels of IL-37 in HCs and the total RA patient cohort at baseline, and after three months of DMARD treatment. (**B**) Plasma levels of IL-37 in drug responders compared to the same patients at baseline (grey dots). (**C**) Plasma levels of IL-37 in drug non-responders compared to the same patients at baseline (white dots). The horizontal lines indicate the median values of the different groups. The difference between the groups was analyzed by Mann-Whitney test and Wilcoxon signed rank tests for unpaired and paired comparisons, respectively.

**Figure 2:**
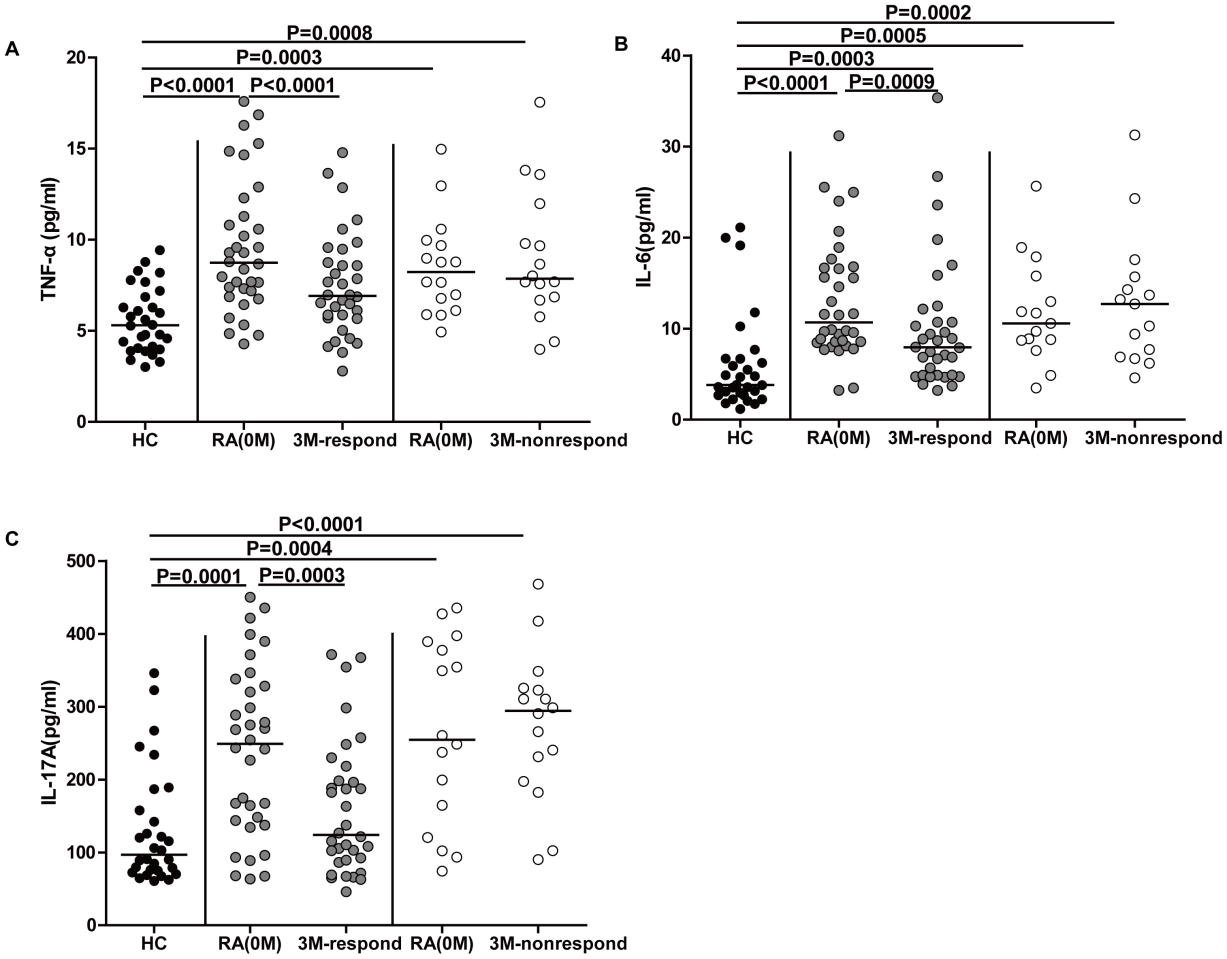
Plasma levels of TNF-α, IL-6 and IL-17A in RA patients. (**A**) Plasma levels of TNF-α in HCs, drug responders, and drug non-responders at baseline and after three months of treatment. (**B**) Plasma levels of IL-6 in HCs, drug responders (grey dots), and drug non-responders at baseline and after three months of treatment (white dots). (**C**) Plasma levels of IL-17A in HCs, drug responders (grey dots), and drug non-responders at baseline and after three months of treatment (white dots). The horizontal lines indicate the median values of the different groups. The difference between the groups was analyzed by Mann-Whitney test and Wilcoxon signed rank tests for unpaired and paired comparisons, respectively.

It has been reported that IL-37 plays an anti-inflammatory role in various diseases [14]. However, the effect of DMARD treatment on IL-37 in RA patients has not yet been established. Therefore, this study assessed the levels of plasma IL-37 in RA patients before and after DMARD treatment, and in comparison to HCs. The plasma levels of IL-37 in the full cohort of RA patients before treatment were significantly higher compared to levels in HCs (Fig. 1A, P<0.0001). However, there was no statistically significant difference between IL-37 levels before and after three months of DMARD treatment for the full patient cohort. Based on the effect of DMARD treatment on the DAS28 score, the 50 RA patients were divided into two groups: 34 patients were determined as being responsive to DMARD treatment, while the other 16 patients were determined to be unresponsive to DMARD treatment. As shown in Fig. 1B, plasma levels of IL-37 decreased in drug-responsive RA patients compared to the same patients at baseline (Fig. 2B, P = 0.0014). However, there was no statistically significant difference between IL-37 levels in RA patients not responding to treatment compared to the same patients at baseline (Fig. 2C).

### Plasma Levels of TNF-α, IL-6 and IL-17A in RA Patients during DMARD Treatment

TNF-α, IL-6 and IL-17A play a crucial role in the progression of RA [3], [4], [7]. In this study, we determined plasma levels of these cytokines in RA patients following DMARD treatment. Consistent with previous studies, plasma levels of TNF-α were significantly higher in both groups of RA patients at base line compared to TNF-α levels in HCs (Fig. 2A, P<0.0001 and P = 0.0003, respectively). The plasma levels of IL-6 were also higher in both groups of RA patients at baseline compared to HCs (Fig. 2B, P<0.0001 and P = 0.0003, respectively). Furthermore, the plasma levels of IL-17A in both groups of RA patients at baseline were also elevated compared to IL-17A levels in HCs (Fig. 2C, P = 0.0001 and P = 0.0004, respectively). Following DMARD treatment for three months, drug responders among the RA patients had lower levels of plasma TNF-α, IL-6 and IL-17A than before treatment (Fig. 2A, P<0.0001; 2B, P = 0.0009 and 2C, P = 0.0003, respectively). In contrast, there was no statistically significant difference between the plasma levels of these cytokines in drug non-responders before and after treatment.

### Correlation of IL-37 with Other Cytokines in RA Patients

**Figure 3:**
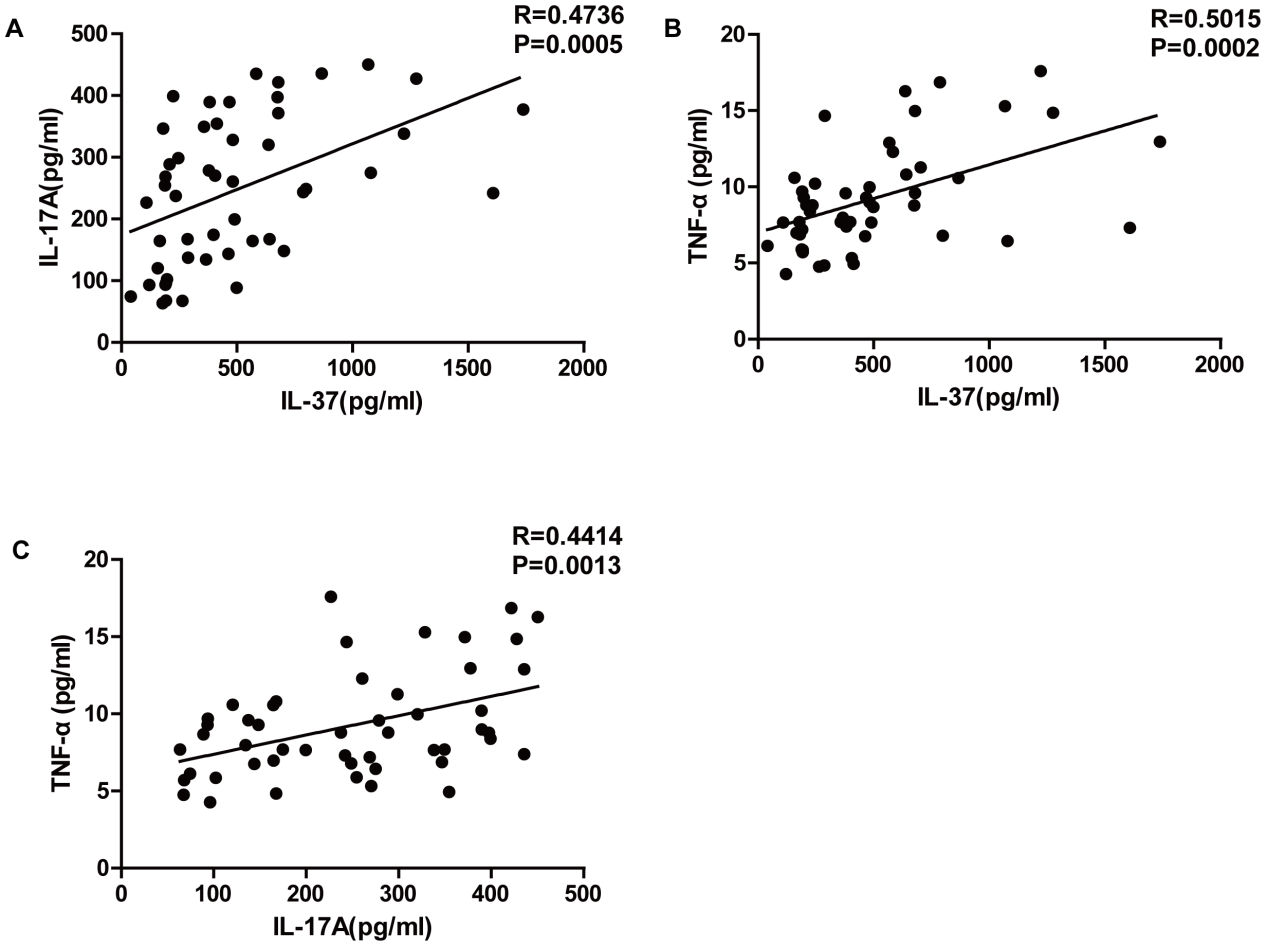
Correlation between IL-37 and other cytokines in RA patients. (**A**) Correlation between IL-37 and IL-17A in plasma of RA patients. (**B**) Correlation between IL-37 and TNF-α in plasma of RA patients. (**C**) Correlation between IL-17A and TNF-α in plasma of RA patients. The relationship between variables was evaluated using the Spearman rank correlation test.

The relationship between IL-37 and pro-inflammatory cytokines in the plasma of RA patients has not yet been investigated. We found that the plasma levels of IL-37 in RA patients were positively correlated with IL-17A (Fig. 3A, R = 0.4736, P = 0.0005) and TNF-α (Fig. 3B, R = 0.5015, P = 0.0002) levels. Additionally, the plasma levels of IL-17A were positively correlated with TNF-α (Fig. 3C, R = 0.4414, P = 0.0013).

### Correlation of IL-37 with Disease Activity in RA Patients

**Figure 4:**
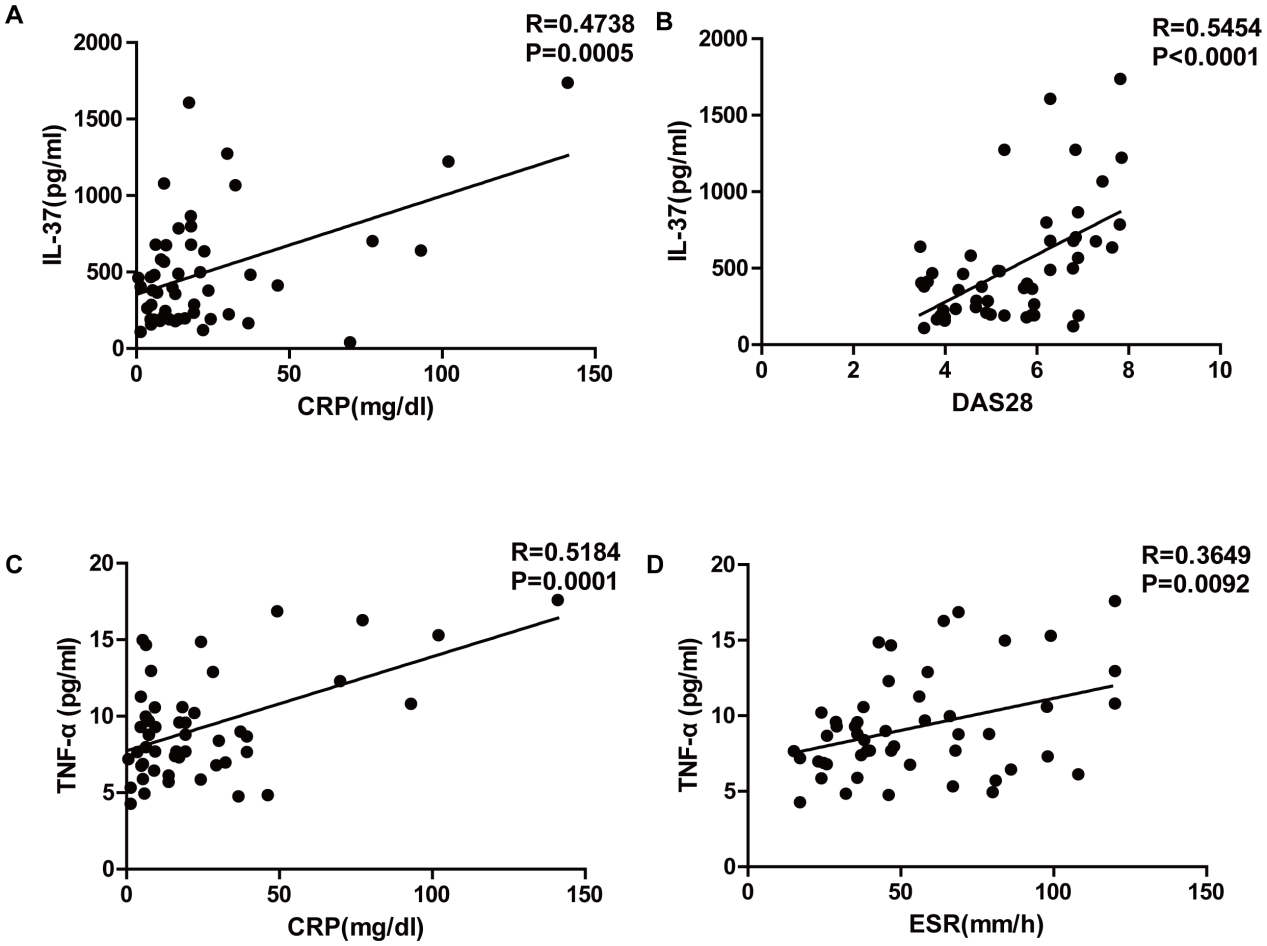
Correlation between plasma levels of IL-37 and disease activity in RA patients. (**A**) Correlation between plasma levels of IL-37 and CRP in RA patients. (**B**) Correlation between plasma levels of IL-37 and the DAS28 score in RA patients. The relationship between variables was evaluated using the Spearman rank correlation test.

We analyzed the relationship between IL-37 and disease activity in RA patients, and found that plasma IL-37 was positively correlated with CRP (Fig. 4A, R = 0.4738, P = 0.0005) and the DAS28 score (Fig. 4B, R = 0.5454, P<0.0001) in RA patients. In addition, plasma TNF-α was positively correlated with CRP (Fig. 4C, R = 0.5184, P = 0.0001) and ESR (Fig. 4D, R = 0.3649, P = 0.0092) in RA patients.

## Discussion

RA is a chronic, systemic inflammatory disease that is characterized by inflammatory cell infiltration, synovial cell proliferation, destruction of cartilage and aberrant post-translational modifications of self-proteins [20]. Numerous cytokines, both pro- and anti-inflammatory, have been detected in inflammatory diseases, and the balance between these opposing cytokine activities has been shown to regulate disease severity [21]. RA is closely associated with increased expression of TNF-α, IL-6, and IL-17A [22]. Therefore, inhibition of TNF-α, IL-6, and IL-17A expression is a promising strategy for the development of novel anti-RA therapies.

The anti-inflammatory mechanism of IL-37 is still not clear. There are currently two hypotheses: either IL-37 is secreted into the extracellular space to inhibit the actions of pro-inflammatory cytokines or their receptors [23], or IL-37 translocates to the nucleus where it interacts with Smad3 to interrupt transcription of pro-inflammatory cytokine genes [10], [12], [13]. IL-37 has been shown to significantly suppress IL-1β-induced expression of IL-1α, IL-8, IL-6, chemokines MIP-2/CXCL-2, MCP-5/CCL-12, and BCA-1/CXCL13, IL-23, IL-1RA, IL-17, IL-18, IFN-γ and TNF-α in several cell types [24]. In contrast, pro-inflammatory cytokines IL-18, IFN-γ, IL-1β and TNF-α can increase synthesis of IL-37 in human peripheral blood mononuclear cells [12], [24]. It has been reported that immunohistochemical staining of the synovial lining in active RA revealed larger amounts of IL-37 compared to HCs [13]. The IL- 37 protein was also detected in plasma of patients with severe Systemic Lupus Erythematosus [25] and in lamina propria macrophages of patients with Crohn’s disease [12]. However, plasma concentrations of IL-37 in RA patients have not yet been reported. Our data show that plasma levels of IL-37 were significantly higher in active RA patients compared to HCs. This suggests that IL-37 may be activated by pro-inflammatory cytokines or other unknown factors in the acute phase of RA.

TNF-α, IL-6 and IL-17A have been considered to be the pivotal cytokines in the pathogenesis of RA, as they are present at biologically significant levels in RA synovial tissue and in synovial fluid [26], [27]. In good agreement, we found that the plasma levels of TNF-α, IL-17A and IL-6 were significantly higher in RA patients than in HCs. Following DMARD treatment, the plasma levels of TNF-α, IL-6 and IL-17A significantly decreased in drug responders. However, there was no significant decrease in cytokine levels in patients who did not respond to DMARD treatment. These data suggest that DMARD treatment in responders resulted in alleviation of the severity of arthritis by down-regulation of pro-inflammatory TNF-α, IL-6 and IL-17A cytokine expression. The plasma levels of IL-37 in drug-responding RA patients also decreased significantly, compared to IL-37 levels before treatment, suggesting that the expression of IL-37 was mostly controlled by pro-inflammatory cytokine levels.

In this study, we found that the plasma level of IL-37 was positively correlated with TNF-α in RA patients. Consistent with our results, a previous study reported that TNF-α could effectively increase the synthesis of IL-37 in PBMCs, using immunoblot analysis of cell lysates [13]. It has been reported that IL-17A is strongly dependent on TNF-α in the early stages of experimental arthritis, and could induce the production of TNF-α at later stages of experimental arthritis [28], [29]. Consistent with these studies, our results indicated that the plasma levels of IL-17A were positively correlated with TNF-α in RA patients with active disease. In addition, the plasma levels of IL-37 were also positively correlated with IL-17A in RA patients. The lack of correlation between IL-6 levels and other cytokines, or the severity of the disease may be attributed to the relatively small sample size.

IL-37 has been reported to be protective against septic shock [12], [13], DSS-induced colitis [30], concanavalin A-induced hepatitis [31], and in an experimental model of ischemia/reperfusion (I/R)-induced hepatitis [32]. These effects are related to the ability of IL-37 to reduce the production of pro-inflammatory cytokines and chemokines [31]. However, the correlation between IL-37 and disease activity in RA patients has not yet been investigated. We found that the plasma level of IL-37 was positively correlated with both CRP and DAS28. This is consistent with the notion that both the levels of IL-37 and the disease activity are determined by the same underlying factors, most likely the levels of pro-inflammatory cytokines.

In conclusion, the plasma levels of IL-37, as well as TNF-α, IL-6 and IL-17A, were found to be significantly increased in RA patients compared to HCs, and decreased in drug responders following DMARD treatment. In addition, plasma levels of IL-37 were positively correlated with the levels of pro-inflammatory cytokines TNF-α and IL-17A, and also with the disease activity in RA patients. Our data are consistent with the notion that the expression of IL-37 is mostly controlled by pro-inflammatory cytokines during the acute phase and the recovery phase of RA. This suggests that IL-37 may be part of a feed-back loop to control inflammation. However, this mechanism does not work effectively to control inflammation during active RA, either because the expression of IL-37 is inadequate, or because the effect of IL-37 is neutralized by unknown factors. These novel findings may provide new insights into understanding the pathogenesis of RA. More detailed studies are warranted to better understand the role of IL-37 in regulating the pathogenesis of RA. Our future studies will determine the role and mechanisms of IL-37 in regulating the pathogenic process of RA.
